# Hand, foot and lip representations in primary sensorimotor cortex: a high-density electroencephalography study

**DOI:** 10.1038/s41598-019-55369-3

**Published:** 2019-12-19

**Authors:** Mingqi Zhao, Marco Marino, Jessica Samogin, Stephan P. Swinnen, Dante Mantini

**Affiliations:** 1 Research Center for Motor Control and Neuroplasticity, KU Leuven, 3001 Leuven, Belgium; 2 Brain Imaging and Neural Dynamics Research Group, IRCCS San Camillo Hospital, 30126 Venice, Italy; 3 Leuven Brain Institute, KU Leuven, 3000 Leuven, Belgium

**Keywords:** Neuroscience, Biomedical engineering

## Abstract

The primary sensorimotor cortex plays a major role in the execution of movements of the contralateral side of the body. The topographic representation of different body parts within this brain region is commonly investigated through functional magnetic resonance imaging (fMRI). However, fMRI does not provide direct information about neuronal activity. In this study, we used high-density electroencephalography (hdEEG) to map the representations of hand, foot, and lip movements in the primary sensorimotor cortex, and to study their neural signatures. Specifically, we assessed the event-related desynchronization (ERD) in the cortical space. We found that the performance of hand, foot, and lip movements elicited an ERD in beta and gamma frequency bands. The primary regions showing significant beta- and gamma-band ERD for hand and foot movements, respectively, were consistent with previously reported using fMRI. We observed relatively weaker ERD for lip movements, which may be explained by the fact that less fine movement control was required. Overall, our study demonstrated that ERD based on hdEEG data can support the study of motor-related neural processes, with relatively high spatial resolution. An interesting avenue may be the use of hdEEG for deeper investigations into the pathophysiology of neuromotor disorders.

## Introduction

The performance of any simple or complex movement requires the coordination of highly specialized brain regions, which dynamically exchange information to support motor planning and execution. The primary somatosensory and motor regions span the precentral and postcentral gyri of the brain, respectively. The primary somatosensory cortex receives neural pulses from the spinal cord, which code somatic stimuli from the contralateral side of the body. In turn, the primary motor cortex generates neural pulses that are sent through the spinal cord to produce contralateral body movements. Somatosensory and motor regions are functionally interdependent, and both crucial for the performance of motor tasks^1^. They are indeed often considered together, and in this case they are referred to as primary sensorimotor cortex. Despite their opposite localization with respect to the central gyrus, both somatosensory and motor representations of different body parts in the primary sensorimotor cortex are orderly arranged from the toe (at the top of the cortex) to mouth (at the bottom)^2^. The amount of sensorimotor cortex devoted to a body part is not proportional to the size of the body part itself, but is rather related to the precision required for somatic sensation and/or movement control.

To investigate the functional organization of the primary sensorimotor cortex, early studies used direct electrical stimulation through implanted electrodes^2^. Although this technique is still used for mapping brain function, the focus of neuroscientific research has shifted toward the use of noninvasive methods. For instance, transcranial magnetic stimulation (TMS) has been used to examine the functional organization of the primary motor cortex^3,4^; however, its spatial specificity may not be sufficient to disentangle the representation of different body parts. Functional magnetic resonance imaging (fMRI) is nowadays the most commonly used noninvasive technique for studying the functional organization of the human brain. By measuring the blood-oxygen-level-dependent (BOLD) signal^5^, fMRI allowed observing changes in brain activity during movement of different body parts, as for instance hand, foot and lips^6,7^. fMRI has shown large reproducibility in mapping of primary motor cortex, as assessed by test-retest analyses^8,9^. Although this technique can provide spatial maps with relatively high resolution, the measured signal is mediated by a slow hemodynamic response. As such, it is not suited to directly investigate motor-related neural processes in the cortex at high temporal resolution.

The two main techniques that can be used for the noninvasive study of neural activity in the human brain are magnetoencephalography (MEG) and electroencephalography (EEG). Through sensors positioned outside the head, MEG and EEG record changes in magnetic fields and electric potentials, respectively, directly produced by neural activity. Different neuronal oscillations can be identified from MEG/EEG signals, based on their frequency. In particular, the power of neural oscillations in the frequency band between 13 and 30 Hz, typically referred to as β rhythm, has been often reported to be modulated by motor performance^10^. Other important neural oscillations are the δ (1–4 Hz), θ (4–8 Hz), α (8–13 Hz) and γ (>30 Hz) rhythms. The task-related power modulation of these rhythms can be quantified in terms of event-related desynchronization/synchronization (ERD/ERS)^11^. ERD is a transient decrease of oscillatory power, typically observed after the onset of an internally or externally paced event, whereas ERS is an increase of oscillatory power, which often occurs after the offset of the event. Neural power changes are typically confined to specific frequency bands, and can be interpreted as changes of local interactions between main neurons and interneurons. Several MEG/EEG studies have been conducted to investigate ERD/ERS in the sensor space, both for real movements^11–13^ and for motor imagery^13–15^. MEG has also been used in combination with source localization techniques for the motor cortex mapping using ERD/ERS^16^. On the contrary, the number of studies using EEG for studying motor-related activity in the source space is very limited^17–19^, possibly due to the intrinsic difficulty in achieving precise EEG source localization^20^. The use of EEG can be of added value as compared to MEG, primarily due to its higher sensitivity to deep sources^21^, the possibility of combining it with brain stimulation and other brain imaging techniques^22,23^, and its suitability for mobile applications (e.g. walking conditions)^24^.

In the last years, novel technical developments have opened the way to the use of EEG as a brain imaging tool^25^. This primarily relates to the introduction of high-density EEG (hdEEG) systems, which yield a higher spatial sampling of scalp potentials^26,27^. Also, novel tools for the removal of artefacts from hdEEG data, the construction of a head model incorporating multiple tissue conductivity and the accurate reconstruction of brain activity in the cortical space have been recently introduced^27^. We used these tools for the analysis of resting state hdEEG data. This permitted us to detect multiple brain networks that were spatially similar to corresponding networks obtained from fMRI data^28^.

The goal of this study is to map the representations of hand, foot, and lip movements in the primary sensorimotor cortex using hdEEG. We aim to demonstrate that source localizations of hdEEG data can yield a spatial accuracy comparable to that of fMRI results, at least for cortical regions. Moreover, by means of hdEEG, we examine the spectral signatures of hand, foot, and lip movement in the primary sensorimotor cortex. In this regard, our hypothesis is that the ERD map in the β band for the different movements spatially matches previously reported fMRI activation patterns. We also expect that oscillations in other frequency bands are differentially expressed during the movement of hand, foot, and lip. This investigation may clarify whether and to what extent hdEEG can be used for detailed analyses of motor-related neural activity in the space and frequency domains. Our findings may support a better understanding of the functional architecture of the motor control system, possibly opening the way for the use of hdEEG in the study of neuromotor disorders and in the definition of targeted/personalized motor rehabilitation protocols.

## Methods

### Experimental design

Sixteen healthy individuals (8 females and 8 males, age 23–39 years) were recruited to participate in the experiment. All the participants were right-handed, and none of them suffered from any brain-related disease/injury or serious medical condition. The experiment procedures were approved by the Ethics Committee Research UZ/KU Leuven (EC Research, reference number s58333), and conducted in accordance with the 1964 Helsinki declaration and its later amendments. Informed consent was obtained from all individual participants included in the study.

The participants were asked to sit on a chair, and to perform specific movements as indicated through a screen in front of them, by using Psychtoolbox-3^29^. The experiment was composed of three runs, in which one of the following movements had to be performed: wrist flexion-extension, foot flexion-extension and lip protrusion-contraction^8,9,30^. Each run contained 30 blocks, with each block consisting of a resting period (6 seconds) and a movement period (6 seconds, self-paced paced at around 2 Hz). The order of the runs was counterbalanced across subjects. Before each run, task instructions were given to the participant. The total duration of the experiment was about 20 minutes for each participant.

### Data collection

We collected 128-channel hdEEG data at 1 KHz sampling rate using an ActiCHamp amplifier (Brain Products GmbH, Germany). With the same amplifier, we also collected 2 electrooculographic (EOG) signals to monitor horizontal and vertical eye movements, and 6 electromyographic (EMG) signals. Three bipolar EMG sensors were positioned to measure the activity of the following muscles: masseter (right), trapezius (right) and splenius capitis (right). Together with the EOG signals, these EMG signals were subsequently used for EEG artefact removal. The remaining three bipolar EMG sensors were placed in correspondence with the extensor carpi radialis longus (right), tibialis anterior (right) and orbicularis oris (right) muscles. These EMG signals were used to extract specific onset triggers for the hand, foot and lip movements, respectively. After EEG/EOG/EMG data collection, we used a Xensor digitizer (ANT Neuro, The Netherlands) to record EEG electrode positions, which were defined according to an equidistant system^31^.

In a separate session, a structural magnetic resonance image of the participant’s head was collected with a 3T Philips Achieva MR scanner (Philips Medical Systems, Best, the Netherlands) using a T1-weighted magnetization-prepared rapid-acquisition gradient-echo (MP-RAGE) sequence. The scanning parameters were TR = 9.6 ms, TE = 4.6 ms, 160 coronal slices, 250 × 250 matrix, voxel size 0.98 × 0.98 × 1.2 mm^3^. The images were later used to generate a volume conduction model for EEG source localization.

### Data analysis

We used a workflow for hdEEG analysis that was previously developed and validated with resting state data^27,28,32,33^ and was extended in the present study to task-related data. The workflow was divided into four mains steps: data preprocessing, head model creation, source-space activity reconstruction, and ERD/ERS analysis.

#### Data preprocessing

The main goal of the data preprocessing step was to correct bad EEG channels, to attenuate artefacts, and to re-reference the EEG signals. To detect bad channels, we used an automated procedure based on two parameters: minimum Pearson correlation of each channel against the other channels in frequency band 1–50 Hz and noise variance in frequency band 200–250 Hz^27^. The bad channels were reconstructed using neighboring channels, as implemented in the FieldTrip toolbox (http://www.fieldtriptoolbox.org)^34^. The EEG signals were then band-pass filtered between 1 and 80 Hz, and artefacts were attenuated by an ICA-based method^35^. In detail, independent components (ICs) were extracted using the fast fixed-point ICA (FastICA) algorithm; then, artefactual ICs were automatically detected based on three parameters: 1) correlation *C*_*p*_ between the ICs and EOG and EMG signals; 2) the coefficient of determination *r*^2^ obtained by fitting the IC power spectrum with a $\frac{1}{f}$ function; and 3) the kurtosis *k* of the IC time-course^27^. In the last stage of data preprocessing, the EEG signals were re-referenced to average reference, which was found to be robust and accurate for hdEEG applications^36^.

#### Head model creation

The use of a realistic head volume conductor model is necessary for accurately estimating the neural sources from EEG data. To create this model from the individual MR image, three steps were followed: electrodes position coregistration, head tissue segmentation, and leadfield matrix calculation^27^. Electrode positions were coregistered to the individual MR space by extracting the headshape from the individual MR image, and using the Iterative Closest Point registration algorithm^37^ for the alignment to the individual scalp profile. The electrode positions were then orthogonally projected over the headshape. Afterwards, we segmented the MR image into 12 tissue classes (skin, eyes, muscle, fat, spongy bone, compact bone, cortical/subcortical gray matter, cerebellar gray matter, cortical/subcortical white matter, cerebellar white matter, cerebrospinal fluid, and brain stem), each characterized by a specific conductivity value set based on previous relevant studies^38,39^. This segmentation step was performed using SPM12 (http://www.fil.ion.ucl.ac.uk/spm/software/spm12) by registering a template image, on which the 12 tissue classes were already defined, to the individual MR space^27,28^. After electrode position coregistration and head tissue segmentation, we calculated the leadfield matrix, which translates the activation of each assumed brain source to scalp electrical potentials, by using a whole-head finite element method (FEM). In detail, hexahedral meshes were generated from the warped 12-layer tissue template. The elements of the mesh corresponding to the gray matter were set to represent possible brain sources. The leadfield matrix was calculated using the Simbio FEM method implemented in the FieldTrip toolbox^34^. The results were finally interpolated into a 6-mm isotropic volumetric grid.

#### Source-space activity reconstruction

Starting from the preprocessed hdEEG signals and the realistic head volume conduction model, brain activity in the source space (encompassing the gray matter) was reconstructed via the exact low-resolution brain electromagnetic tomography (eLORETA) method^40^. Since the activity of each voxel in the source space had three dimensions (x, y, z), we obtained a single time-course by selecting the first principal component, calculated using singular value decomposition.

**Figure 1:**
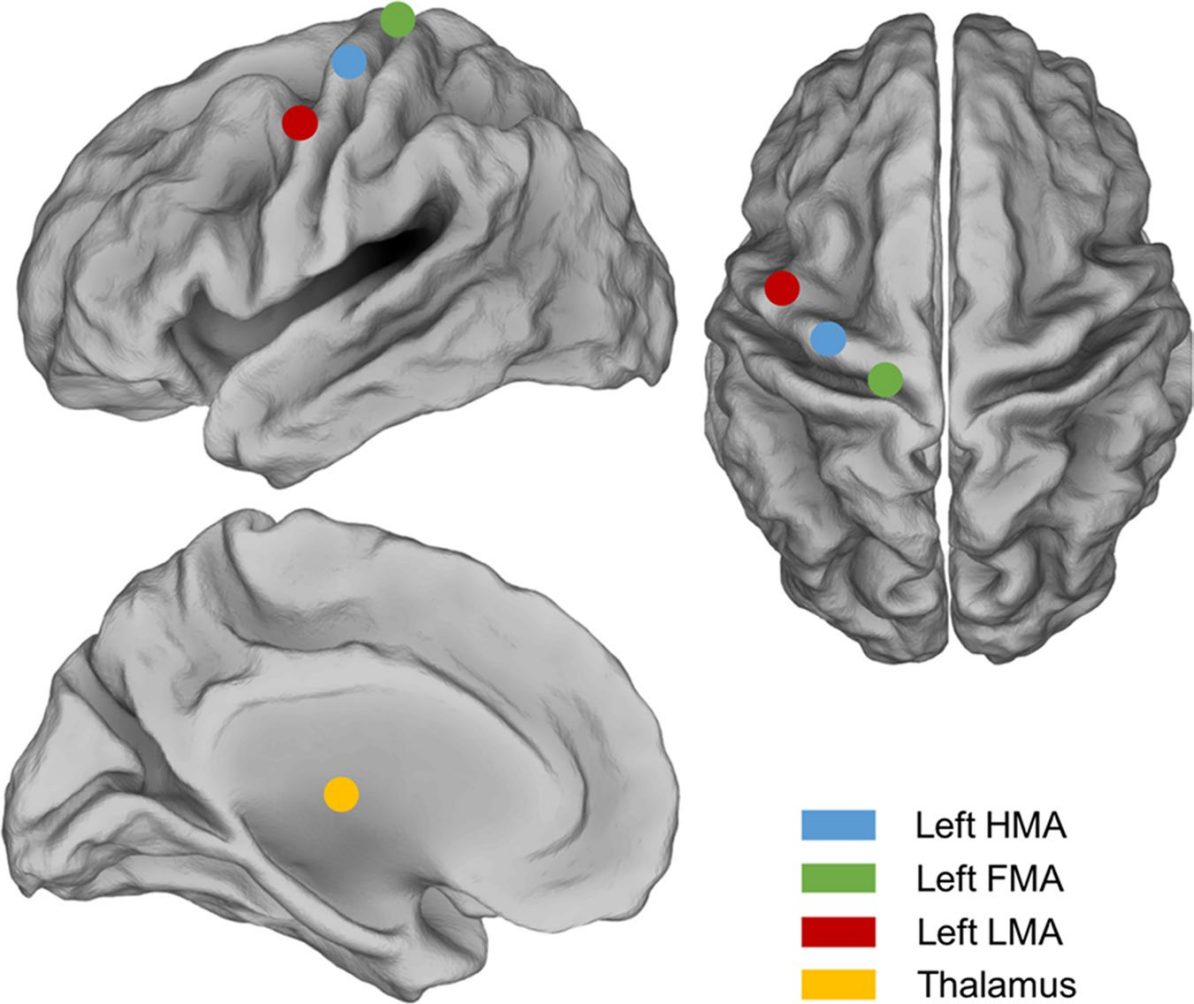
Position of the ROIs for the analysis of motor-related brain activity. The ROIs that were used to assess ERD/ERS during hand, foot and lip movements, are indicated in light blue, green, and red, respectively. The ROIs for the left hemisphere are shown over a cortical representation, both in lateral and dorsal views. The ROIs in the right hemisphere are symmetrically to the ones in the left hemisphere. HMA: hand movement area (MNI coordinates [+/−44, −17,49]); FMA: foot movement area (MNI coordinates [+/−24, −34, 62]); LMA: lip movement area (MNI coordinates [+/−39, 1, 56]; Thalamus (MNI coordinates [−9, −12, 10])).

The ROIs chosen for time-frequency ERD/ERS analysis were defined based on the activations reported in relevant fMRI studies^8,9^. These corresponded to the hand (HMA), foot (FMA) and lip movement (LMA) areas in the primary sensorimotor cortex, for both hemispheres. A thalamic ROI was included as well, to test for the sensitivity of hdEEG in detecting the activity of subcortical sources during movement^41^ (Fig. 1). For each ROI, the MNI coordinates were transformed to individual space, and the voxels within a sphere with 6 mm radius were selected. A representative ROI signal was obtained by calculating the first principal component of the time courses from the ROI voxels.

#### Corticomuscular coherence analysis

Corticomuscular coherence analysis^42^ was performed using the EMG signal *x(t)* recorded during movement performance, in combination with neural activity *y(t)* reconstructed for cortical ROIs. In particular, we calculated global corticomuscular coherence *C* in the band between *f*_*min*_ = 1 Hz and *f*_*max*_ = 80 Hz using the following formula:

1 $$C=\frac{1}{{f}_{max}-{f}_{min}}{\int }_{{f}_{min}}^{{f}_{max}}\frac{{|{G}_{xy}(f)|}^{2}}{\,{G}_{xx}(f){G}_{yy}(f)}$$

where *G*_*xy*_(*f*) is the cross-spectral density between *x(t)* and *y(t)*, and *C*_*xx*_(*f*) and *C*_*yy*_(*f*) are the autospectral densities of *x(t)* and *y(t)*, respectively.

#### ERD/ERS analysis

Frequency-dependent modulations of brain regions were assessed by conducting an ERD/ERS analysis on reconstructed neural signals. We first performed an ERD/ERS analysis for selected ROIs, and we then calculated ERD/ERS maps.

For each signal, corresponding either to a single voxel or to a ROI, a time-frequency analysis was performed by means of short-time Fourier transform, with a moving window of 1 second. Specifically, we generated a spectrogram for the frequency range 1–50 Hz, at steps of 1 Hz, and with temporal resolution equal to 10 ms. The spectrogram was epoched with a time window [−3s, +3 s] centered over the movement onsets time. For each of the three movement conditions, the spectrogram epochs were averaged. As the last step, the ERD/ERS intensity was calculated, for each frequency and time, using the following formula:

2 $$ERS/ERD(f,t)=\frac{P(f,t)-{P}_{B}(f)}{{P}_{B}(f)}\times 100 \% $$

where *P*(*f, t*) is the power at a given frequency and time, and *P*_*B*_(*f*) is the average power in the pre-movement period (baseline, [−3s, 0 s]). ERD/ERS was quantified for the θ (4–8 Hz), α (8–13 Hz), β (13–30 Hz) and γ (30–50 Hz) bands. These frequency bands were defined based on previous studies^43^. The δ band (1–4 Hz) was not considered for this analysis, as it might be contaminated by low-frequency movement artefacts. The post-stimulus interval for the calculation of ERD was in the interval [0 s, +2 s] with respect to movement onset. A repeated-measure two-way analysis of variance (ANOVA) was run to test the influence on the ERD/ERS intensities of motor task and cortical ROI as main factors, as well as of their interaction.

Using the same procedure used for the ROIs, we performed a time-frequency analysis on all voxels in the source space. By averaging the time-frequency maps in the time/frequency intervals used for the ROI analysis, we generated ERD spatial maps for hand, foot and lip movements, respectively. The volumetric images, obtained in individual space, were transformed to MNI space by applying a non-rigid deformation calculated using the T1-weighted MR image of the participant’s head.

Group-level analyses were performed on the ERD spatial maps by using a random-effect analysis. Specifically, a t-test across participants was calculated for each of the three movement conditions in each frequency band. The threshold for statistical significance was set to p < 0.05, corrected for multiple comparisons using false discovery rate (FDR). We also compared the ERD maps across motor tasks and frequency bands. This analysis was conducted by calculating the spatial correlations between unthresholded maps.

## Results

**Figure 2:**
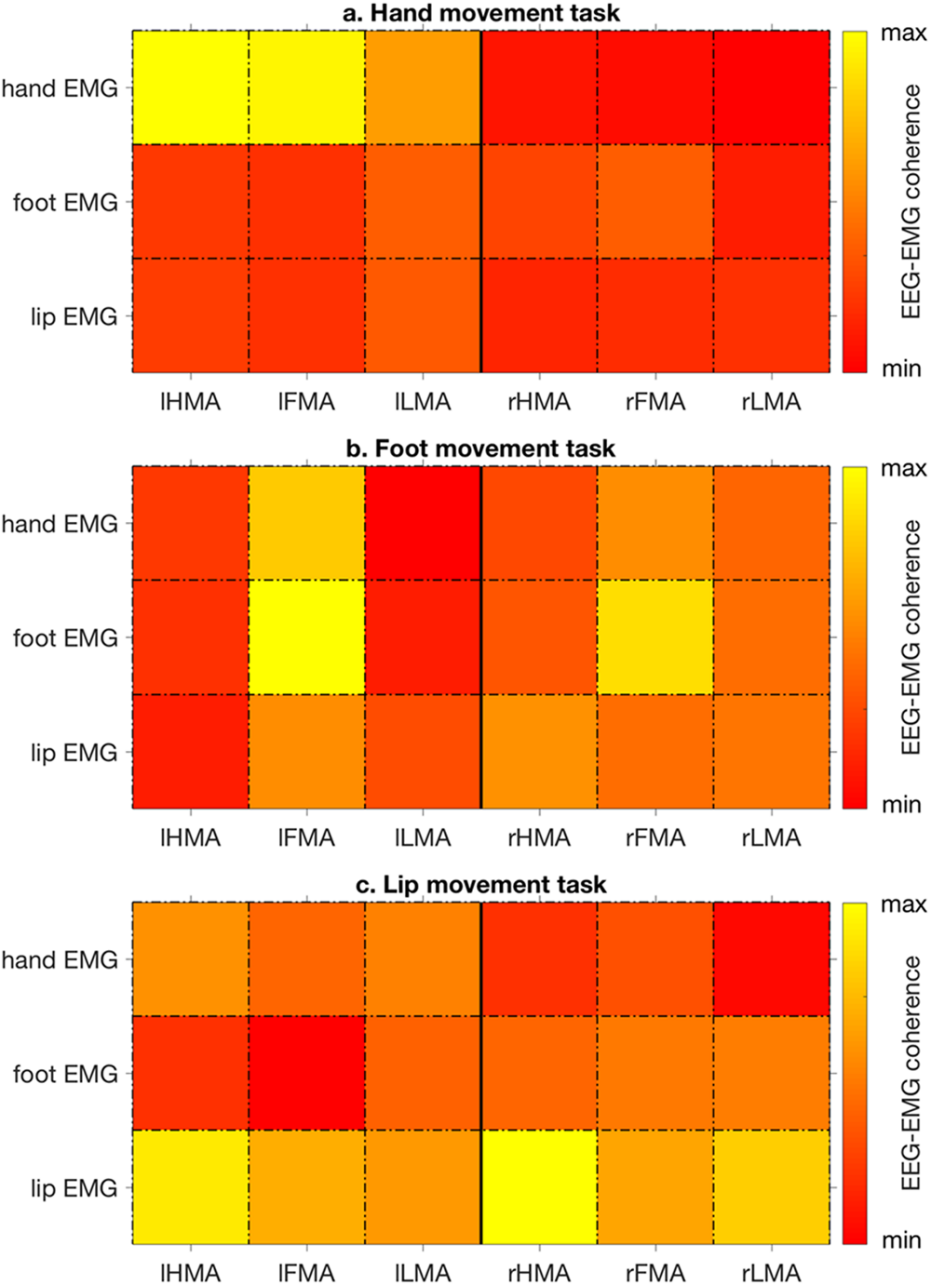
Patterns of corticomuscular coherence during movement performance. Coherence was evaluated for cortical ROIs in both hemispheres during the (**a**) hand, (**b**) foot and (**c**) lip movement tasks, respectively. For each panel, the vertical axis indicates the EMG channel analyzed, and the horizontal axis indicates the ROI for which coherence was measured. lHMA: left hand movement area; lFMA: left foot movement area; lLMA: left lip movement area; rHMA: right hand movement area; rFMA: right foot movement area; rLMA: right lip movement area.

**Figure 3:**
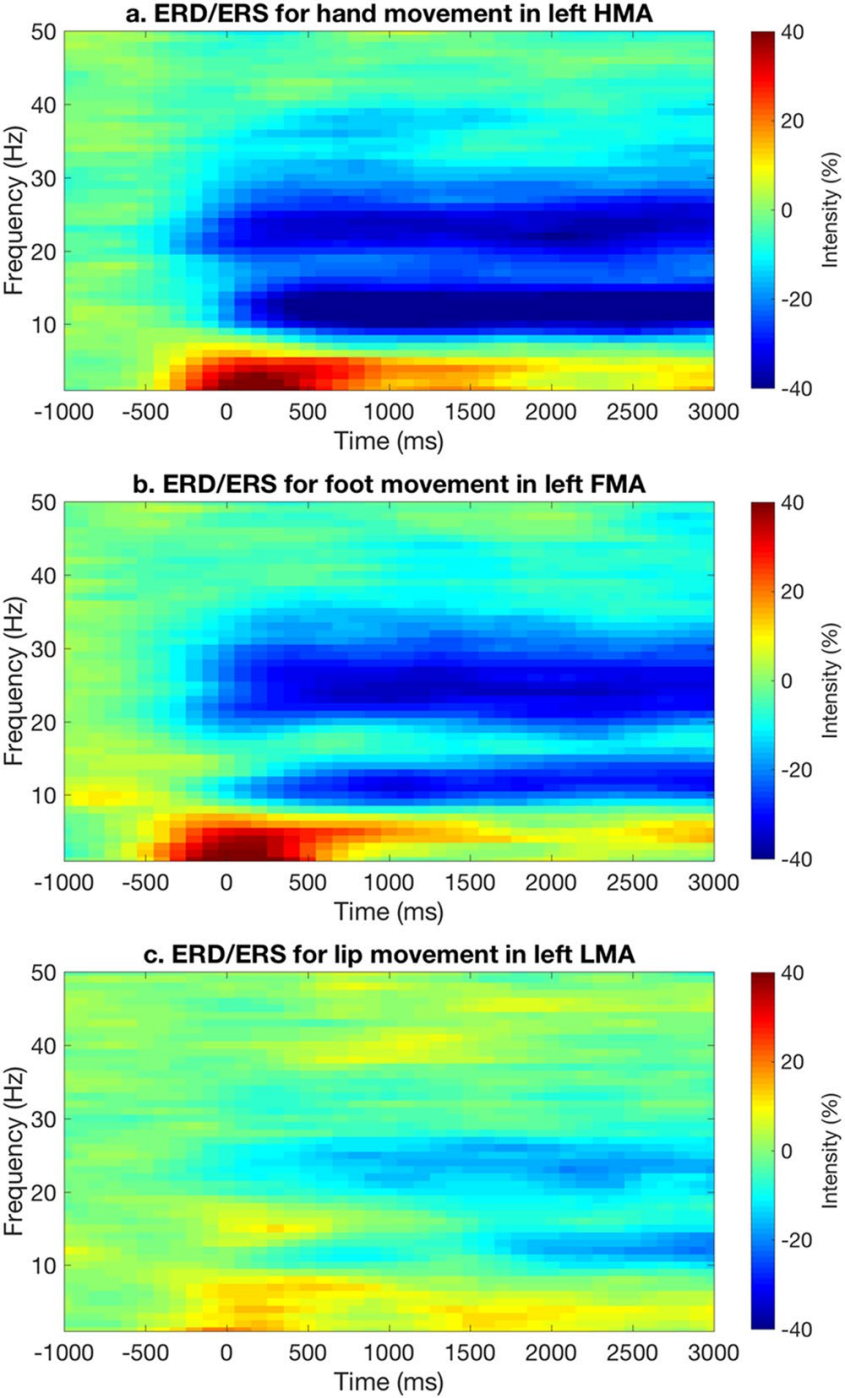
Group-level ERD/ERS analysis for selected ROIs. Time-frequency analyses were conducted for hand movements in left HMA, foot movements in left FMA and lip movements in LMA, respectively. The corresponding plots obtained from individual subjects were averaged, to obtain group-level results.

We expected to observe modulations of neuronal activity in motor-related brain regions during task performance. To test for that, we first conducted a corticomuscular coherence analysis for HMA, FMA and LMA in both hemispheres. We found that the coherence between neural and muscular activity was modulated by the specific movement that was performed (Fig. 2). During movement of the right hand, coherence was much stronger in the left than the right hemisphere, and maximum in left HMA. Likewise, coherence during foot movement showed the strongest value in left FMA, but was relatively large also in the adjacent areas left HMA and right FMA. Coherence during movement of the lips was not strongly lateralized, and with largest values in bilateral HMA rather than in bilateral LMA. To examine frequency-specific neural power modulations, we then conducted an ERD/ERS analysis for HMA, FMA and LMA, respectively (Fig. 3). We first focused on ROIs in the left cerebral hemisphere, as the participants were required to move the right hand and the right foot. For all the three kinds of movements, we observed an ERD in α and β frequency bands, and to a lesser extent in the γ band (Fig. 3). These modulations of neural power started around the movement onset, and remained relatively stable during motor task execution. On the other hand, we also found a strong but transient ERS in the δ band at movement onset. This δ-ERS was not investigated further, as the frequency and timing of the response may be consistent with movement artefacts. We observed a clear, but much less intense α- and β-ERD for the lip than for the hand and foot movements. A slight power increase was also found in the γ band, specifically between 40 Hz and 50 Hz. This ERS could be partially related to residual muscular artefacts in the EEG data.

**Figure 4:**
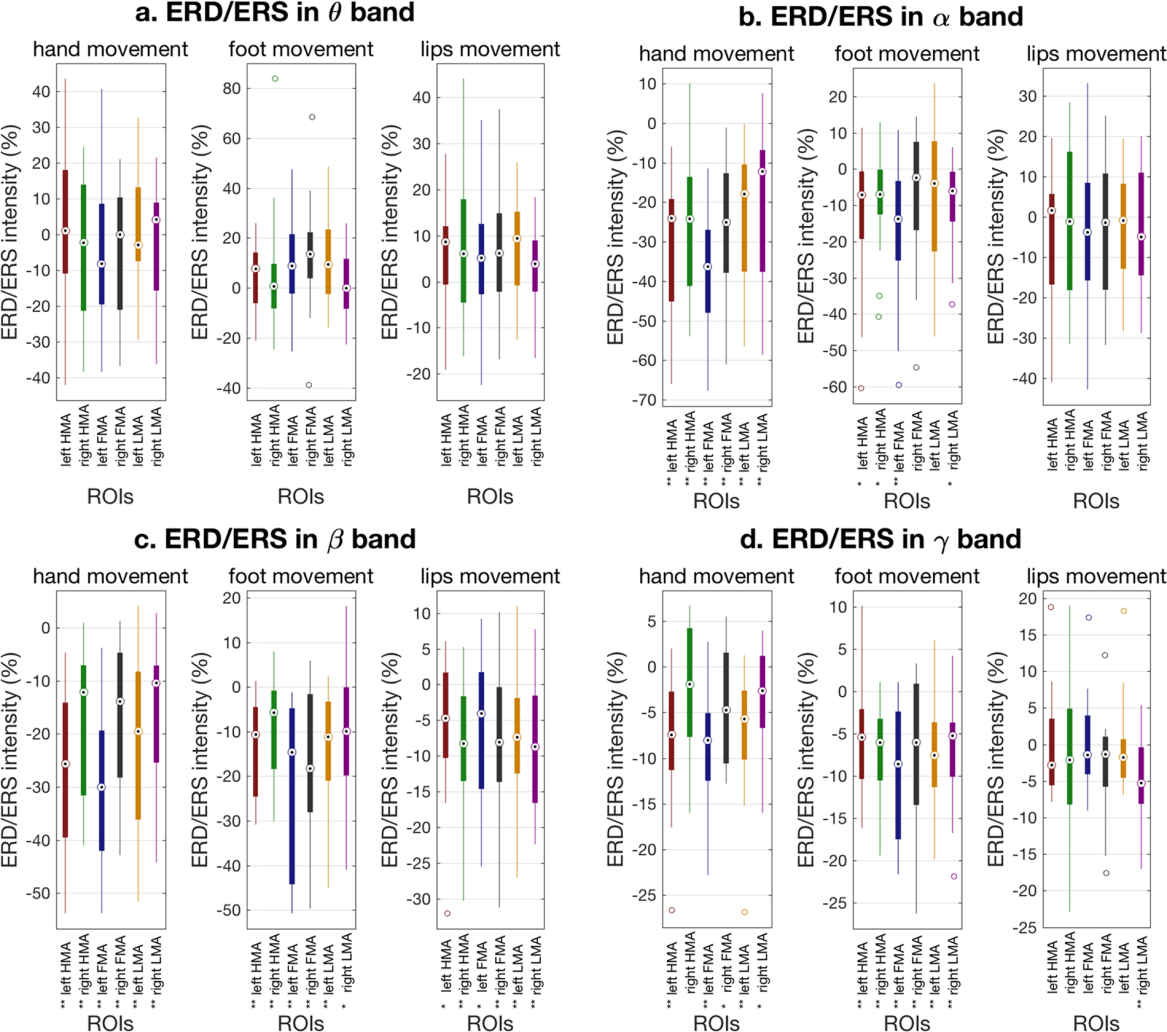
Analysis of ERD intensity across cortical ROIs and motor tasks. The box plots show the ERD intensity for different ROIs and motor tasks in (**a**) θ, (**b**) α, (**c**) β and (**d**) γ bands, respectively. A one-sample t-test was performed on the ERD/ERS intensity for each ROI and task, to test the significance of ERD (*p < 0.05; **p < 0.01). For each motor task, a strong ERD appeared on corresponding ROIs in α, β, and γ bands, which explains the high interaction between motor task and ROI in these bands.

We extended our analysis to the ROIs related to hand, foot and lip movements, but in the right hemisphere. We then statistically evaluated the modulations of ERD intensity values for each motor task and each ROI by using a repeated-measure two-way ANOVA (Table 1). Such an analysis allowed us to examine not only the effects of motor task and ROI as main factors to explain ERD intensity, but also their interaction. The ANOVA revealed that the ERD intensity differed significantly from task to task in the α (p = 4.852e-06), β (p = 7.367e-06) and γ (p = 0.002) bands. The ERD intensity was significantly different across ROIs in α (p = 2.628e-4) and β (p = 0.009) bands. Interestingly, we also found an interaction between tasks and ROIs for the β (p = 1.318e-06), γ (p = 0.003) and α (p = 0.046) bands. When we examined more in detail the ERD values across motor tasks and ROIs (Fig. 4), we observed that: 1) there was no significant ERD in the θ band; 2) a similar pattern of α-ERD values across ROIs was found for hand and foot movements. 3) there was a significant β-ERD for all the three motor tasks. For the hand movement, the left HMA and the left FMA had the largest ERD; the foot movement induced the largest ERD in the left FMA; for the lip movement, the strongest ERD was observed in the right LMA, but an ERD was also found in the left LMA 4) for the γ band, there was a similar pattern of results as for the β band, but the ERD intensities were relatively lower.

**Table 1:** Two-way ANOVA of ERD/ERS intensity across motor tasks, ROIs, calculated for different frequency bands.

Factor\band	DF		θ band	α band	β band	γ band
Motor task	2	F	2.012	18.913	17.982	7.900
		P	0.151	**4.852e-06	**7.367e-06	**0.002
ROI	5	F	1.062	5.416	3.290	0.494
		P	0.388	**2.628e-04	**0.009	0.780
Motor task x ROI	10	F	1.363	1.925	5.303	2.813
		P	0.203	*0.046	**1.318e-06	**0.003

F-scores and P-values of the main factors (Motor task and ROI) and their interaction are displayed for each of the frequency bands. P-values with p < 0.05 and p < 0.01 are marked by “ *” and “ **”, respectively.

**Figure 5:**
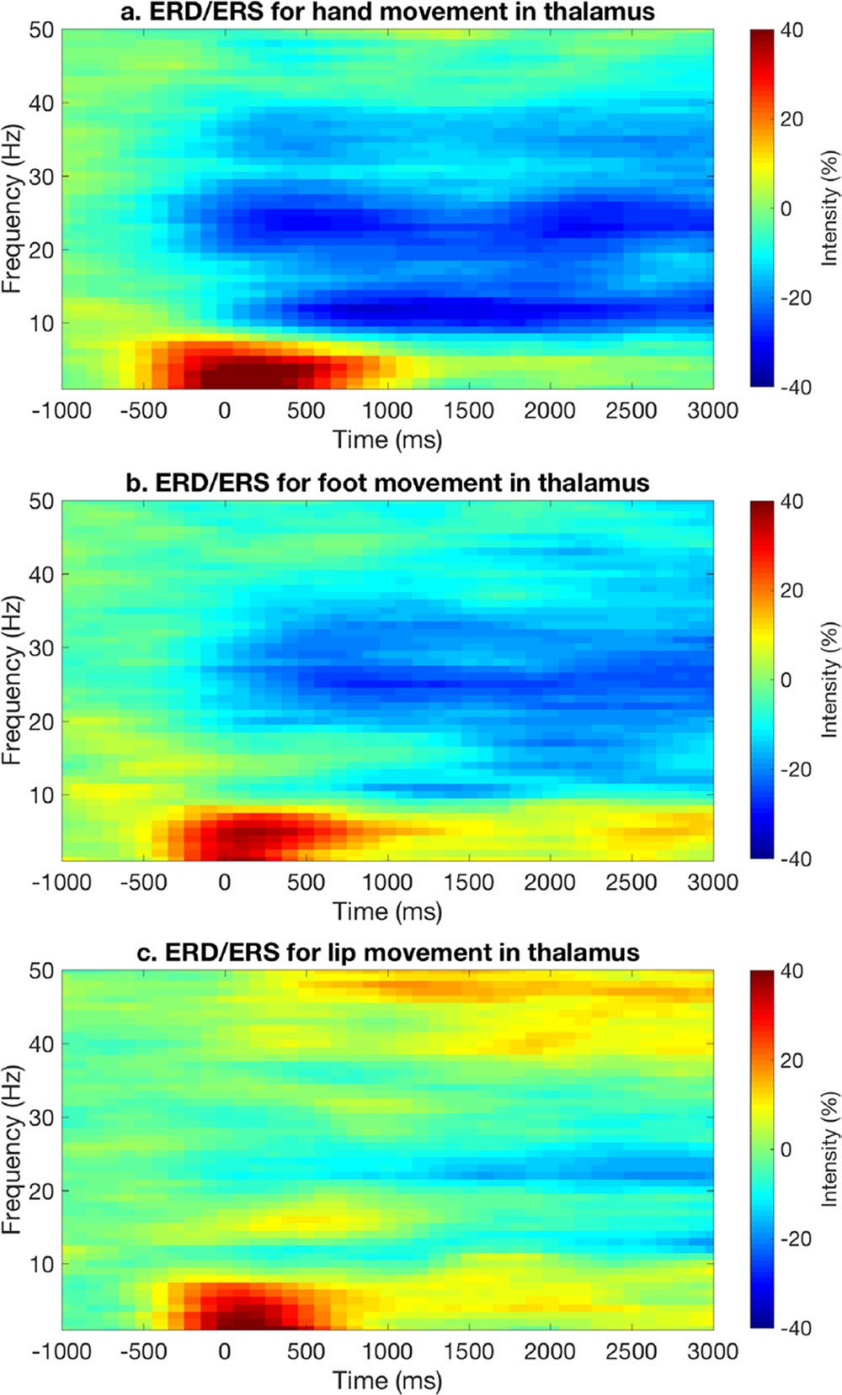
Group-level ERD/ERS analysis for the thalamic ROI. Time-frequency analyses were conducted for (**a**) hand, (**b**) foot and (**c**) lip movements, respectively. The corresponding plots obtained from individual subjects were averaged, to obtain group-level results.

The detection of motor-related power modulations in cortical ROIs for different frequency bands brought us to investigate if it was possible to detect a similar pattern of results also in the thalamus. ERS/ERD results for the thalamic ROI were in line with that of the cortical ROIs, although less prominent (Fig. 5). In particular, clear β-ERD and γ-ERD were observed in the thalamus for the hand and foot movements, but not for the lip movement.

**Figure 6:**
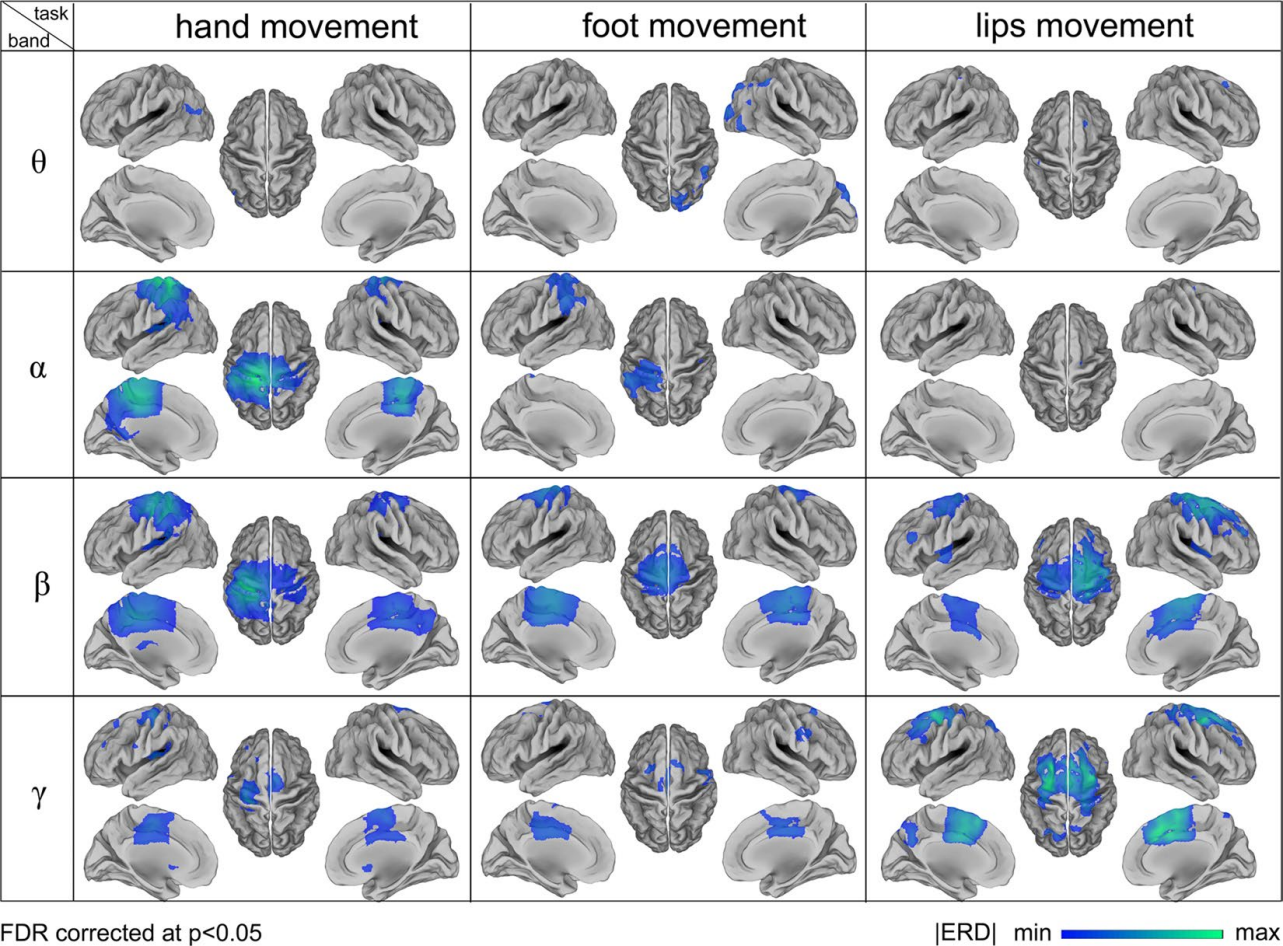
Source-space ERD maps for different frequency bands and motor tasks. The ERD maps, which are represented over a cortical surface in lateral, medial and dorsal views, are thresholded at p < 0.05, corrected for multiple comparisons using FDR. ERD maps in the β and γ band have relatively larger task-related spatial specificity compared to θ and α bands. In β band, ERD maps for the hand and foot movements peaked around left HMA and left FMA respectively, whereas the ERD map for the lips movement showed less reliable results.

After examining motor-related modulations of neural power in predefined ROIs, we also investigated ERD during hand, foot and lip movements at the whole-brain level. More specifically, we calculated ERD maps for θ, α, β and γ bands, respectively (Fig. 6). In general, ERD in the β and γ band had relatively larger task-related spatial specificity in the primary sensorimotor cortex compared to θ and α bands. The α-ERD map for the hand movement covered most of the primary motor area in the left hemisphere and peaked close to the cortical location putatively related to hand movements. On the other hand, the peak locations of α-ERD maps for foot and lip movements were relatively far from the expected locations. The β-ERD maps for the three motor tasks showed spatial patterns more similar to those that could be expected based on fMRI studies. However, the β-ERD map for the lip movement contained regions that are more dorsal as compared to fMRI maps. The γ-ERD maps of the three motor tasks appeared to be more spatially specific than those of β-ERD maps. Overall, ERD maps showed only a minimal contribution of the thalamus, probably due to the lower signal intensity in subcortical regions.

**Figure 7:**
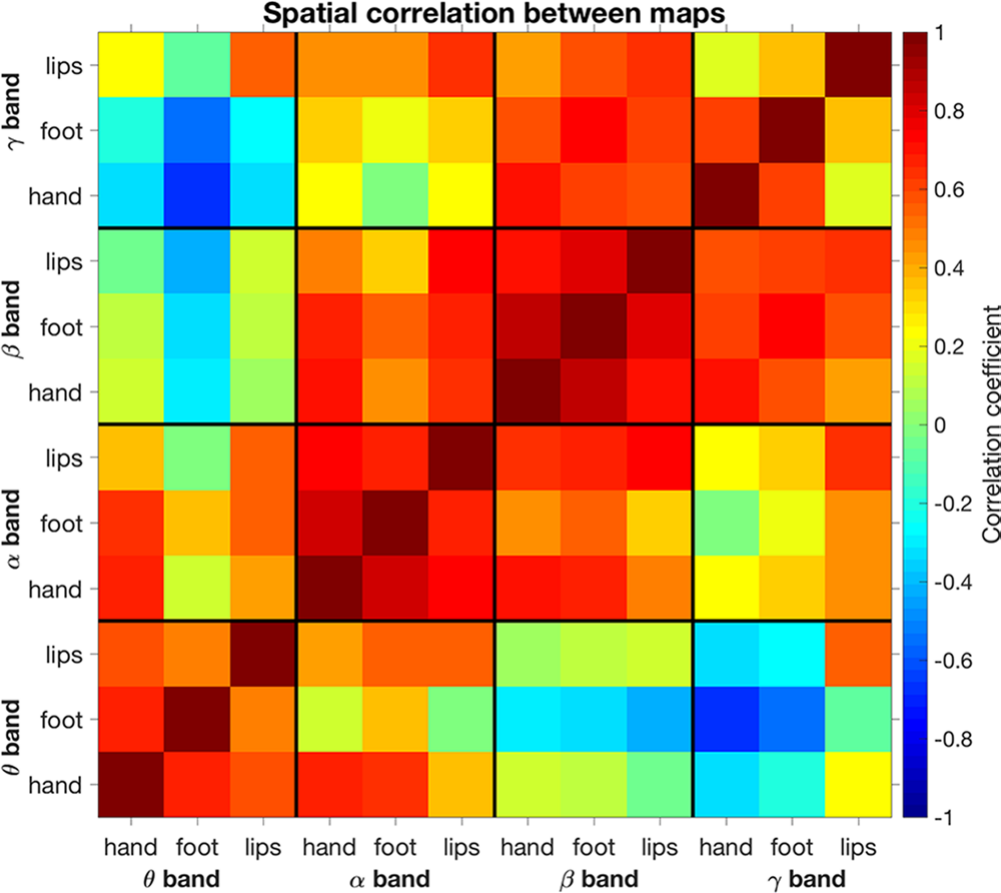
Spatial correlations between ERD maps, calculated for different motor tasks and frequency bands.

To further compare the spatial pattern of ERD across motor tasks and frequency bands, we calculated spatial correlations on unthresholded maps (Fig. 7). In general, relatively high correlation values were found between ERD maps for neighboring frequency bands (e.g., θ and α bands). Interestingly, we observed very low correlation values between ERD maps for θ and β bands, and even negative values between ERD maps for θ and γ bands. Overall, the spatial correlation between different tasks with corresponding bands was significantly larger than that between different frequency bands with corresponding motor tasks (unpaired t-test, p = 0.042). When we examined the correlations between motor tasks separately for each frequency band, we found that the γ band had the lowest values.

## Discussion

The main goal of this study was to map the representations of hand, foot, and lip movements in primary sensorimotor cortex using hdEEG, and to investigate their spectral signatures. To this end, we relied on our recent developments for obtaining accurate reconstructions of neural activity in the cortex from hdEEG recordings^27,28^. We generated ERD maps for different frequency bands, ranging from θ to γ^11^. Our results showed that motor-related ERD/ERS activity could be mapped in the primary sensorimotor cortex, as previously done with fMRI^7,8,44^. Particularly, the β- and γ-ERD maps for hand and foot movements were found to be spatially specific. The activated regions covered a large part of the primary sensorimotor cortex, but the peak locations were consistent with those previously reported in TMS and fMRI studies^8,9,30^. The ERD maps for the lip movement task, however, were not fully consistent with previous studies^8,45^. It may be argued that further methodological work is necessary to increase the sensitivity and accuracy of hdEEG-based analyses when neural sources are relatively less strong. We will more extensively comment on the points mentioned above in the following sections.

Recent technological developments have led to an increased interest of the neuroscientific community in the use of EEG as a brain imaging tool^25^. Critically, combining high-density electrode montages with accurate head models has permitted more precise source localizations in the cortex^27,28^. ERD/ERS has been considered a promising functional brain imaging approach for years^46^. However, ERD/ERS studies often analyzed time-frequency features of single channels or relied on topography maps in the sensor space. Accordingly, the spatial characteristics of ERD/ERS were either neglected or underestimated. The finer spatial specificity brought by the use of hdEEG-specific methods is critical for an accurate mapping of motor-related modulations of neural activity. In this respect, our results support the idea that hdEEG can be used for resolving brain dynamics with relatively high spatial resolution. As such, hdEEG could be used as an alternative to fMRI for functional brain imaging, with the additional benefit of directly measuring brain activity. A possible downside, however, is the more limited sensitivity than fMRI to brain activity in subcortical regions.

Notably, the use of hdEEG permits, as is also the case for MEG, to investigate the contribution of different neural oscillations across brain regions. For instance, we found in our study that the β- and γ-ERD maps, and much more than the θ- and α-ERD maps, showed a topology compatible with the representations of hand, foot and lip movements in the primary sensorimotor cortex. Particularly, our analyses revealed that the spatial correspondence between bands for the same motor task was substantially lower than that observed between different tasks for the same band. This suggests that rich information about motor processes can be disclosed by frequency-based analyses. We found that ERD maps for neighboring bands were similar. ERD maps for different frequency bands, such as θ and γ, even had negative spatial correlation. This result may not be fully surprising, since different neuronal oscillations are unevenly distributed across the cortex^43^.

A large body of studies indicated that motor-related brain activity is primarily represented by neural oscillations in the β band. For instance, electrocorticography studies showed that β-ERD was more spatially specific than that of α-ERD^47^. Also, sensor-based EEG studies supported this idea that β-ERD/ERS reflects an increase/decrease of excitability during movement^46^. We found the β-ERD to be stronger in the contralateral hand and foot region of the primary sensorimotor cortex while the participants were moving the hand or foot, respectively^11^. This is consistent with previous findings obtained using TMS and fMRI^8,9^. Among the three motor tasks used in the study, the symmetrical lip protrusion-contraction is the one that does not require fine movement control, and is therefore expected to produce weaker ERD/ERS. In turn, the lower signal-to-noise ratio (SNR) might explain the difficulty in identifying lip movement representations in the primary sensorimotor cortex. Interestingly, we also observed that the γ-ERD map was spatially similar to the β-ERD map. This finding is however not consistent with other studies reporting a γ-ERS rather than a γ-ERD during movement, with primary frequency being typically around 40 Hz^11,47^. A possible reason for this contradictory finding could be in the choice of the specific time interval and frequency range for the calculation of the ERD. Overall, the finding that both β- and γ-ERD contain information about the specific motor task being performed is particularly interesting in the context of brain computer interface (BCI) research. We suggest that the comparison and/or potential integration of β- and γ-band activity in the source space may enhance the decoding of motor tasks^48–50^.

There are some limitations in the present study, which need to be mentioned. First, EEG source localization is influenced by the choice of the methods used for the solution of forward and inverse problems. Further investigations should be conducted to ensure that the most suitable solutions for analyzing motor-related EEG data are used. Second, the number of participants included in the study limits the generalizability of our results. It should be considered, for instance, that the ERD results for the lip movement may be influenced not only by the possibly weak neural modulations, but also by the limited sensitivity associated with a relatively small sample size. Third, we did not directly compare the ERD results from hdEEG data with fMRI activation results obtained in the same participants. In future studies, simultaneous EEG-fMRI data may be acquired during performance of motor tasks, such that it would be possible to conduct cross-modal comparisons not only at the group- but also at the single-subject level. Fourth, we examined corticomuscular coherence and neural power modulations during task performance. It would also be interesting to examine patterns of task-related connectivity in the brain, by using more complex experimental paradigms^51,52^.

To summarize, we investigated the spatial and spectral features of neural activity elicited by the performance of hand, foot, and lip movements by using hdEEG. Modulations of motor-related neural oscillations were observed in multiple bands. ERD in β and γ bands showed relatively high spatial specificity, with spatial maps well aligned with previous fMRI results. As for lip movements, we had less reliable ERD results. In this case, the neural power modulations might be weaker than for hand and foot movements, given that less fine movement control was required. Overall, our results confirm that hdEEG is a suitable technique to resolve motor-related brain activity with relatively high spatial and spectral resolution. Our future work will focus on methodological developments necessary for the use of hdEEG in clinical settings, and to enable a better understanding of the physiopathological mechanisms underlying neuromotor disorders^53^. This may also contribute to the definition of targeted/personalized motor rehabilitation protocols in the future.
